# Association of Insulin Resistance, Sarcopenia, and Risk of Cardiovascular Disease: Findings From the China Health and Retirement Longitudinal Study

**DOI:** 10.2196/80115

**Published:** 2025-12-31

**Authors:** Huilin Liu, Sijing Li, Ximin Zhang, Wenjie Long, Huili Liao, Lu Lu, Shihao Ni, Zhongqi Yang

**Affiliations:** 1 Department of Geriatrics, First Affiliated Hospital of Guangzhou University of Chinese Medicine, No. 12 Airport Road, Baiyun District, Guangzhou, Guangdong Province, 510405, China, 8613688867618; 2 Lingnan Medical Research Center, Guangzhou University of Chinese Medicine, Guangzhou, Guangdong Province, China

**Keywords:** sarcopenia, insulin resistance, cardiovascular disease, China health and retirement longitudinal study, aging

## Abstract

**Background:**

Cardiovascular disease (CVD) is the main cause of death in middle-aged and older people in China. The interplay between sarcopenia and insulin resistance (IR) in driving CVD risk has not been fully understood, particularly regarding sarcopenia severity and IR heterogeneity.

**Objective:**

This study aimed to investigate the relationship between IR and sarcopenia and the risk of new-onset CVD.

**Methods:**

Using data from the China Health and Retirement Longitudinal Study (CHARLS). Cox proportional hazards models were used to assess associations of sarcopenia status (nonsarcopenia, possible sarcopenia, sarcopenia, and severe sarcopenia) and 6 IR indices (triglyceride-glucose, TyG; TyG-BMI; TyG-waist circumference; TyG-waist-to-height ratio; triglyceride/high-density lipoprotein cholesterol, TG/HDL-C; and metabolic score for insulin resistance, METS-IR) with incident CVD. Additive and multiplicative interaction analyses and subgroup analyses by age and sex were performed. Receiver operating characteristic analysis was used to determine clinically relevant cutoffs.

**Results:**

In this study, during a median 9-year follow-up, we included 5514 middle- and older-aged (≥45 y) residents, of whom 550 presented with CVD incidence. Participants with possible sarcopenia and high IR exhibited 1.24‐1.85-fold higher CVD risk versus nonsarcopenia and low-IR counterparts (*P*<.05) after adjustment for potential confounders. While TyG-BMI and TyG-waist circumference were the strongest independent predictors, formal interaction analysis revealed that the TG/HDL-C ratio and METS-IR demonstrated the most consistent synergistic effects with possible sarcopenia (relative excess risk due to interaction=0.139 and 0.074, respectively). In subgroups of different ages and sexes, the combination of IR and sarcopenia is associated with the highest risk of CVD. Receiver operating characteristic analysis provided clinically applicable cutoffs for these indices, including TG/HDL-C ≥2.09 and METS-IR ≥34.26.

**Conclusions:**

We found that IR and sarcopenia, especially early-stage sarcopenia, synergistically increase the incidence of CVD in older adults. These findings advocate for dual-targeted CVD interventions (muscle preservation and IR mitigation) in aging societies, particularly during the transitional phase of possible sarcopenia.

## Introduction

Aging populations represent a significant public health challenge globally, especially in Asian countries transitioning toward higher proportions of older individuals [1]. Previous research has indicated that age is a primary risk factor for cardiovascular disease (CVD), a leading cause of mortality in older adults. The global burden of CVD is substantial, with deaths rising from 13.1 million in 1990 to 19.2 million in 2023, of which ischemic heart disease and stroke constituted 81.8% [2]. This trend is markedly evident in China, where CVD-related deaths surged by 89.1%, from 2.4 million to 4.6 million, between 1990 and 2019 [3]. Critically, this burden is projected to rise continuously with the rapid growth of the aging population [4]. The ADDIN EN.CITE [5-7] susceptibility to CVD among older adults is further exacerbated by biological factors associated with aging, including hormonal changes, immunosenescence, impaired autophagy, oxidative stress, and mitochondrial dysfunction [8]. Despite addressing well-established CVD risk factors, such as hypertension, diabetes, smoking, and obesity, CVD remains a main cause of mortality and disability among middle-aged and old-aged populations in China, indicating the presence of additional, less recognized risk factors. This study focuses on 2 such potential contributors: insulin resistance (IR) and sarcopenia.

IR is a critical pathological process associated with type 2 diabetes (T2D) and is linked to hypertension and coronary heart disease (CHD), contributing to CVD risk by increasing vascular stiffness [9-11]. However, standard methods for diagnosing IR are complex and time-consuming [12], prompting interest in alternative indices derived from biochemical markers and anthropometric factors, such as triglyceride-glucose (TyG) [13] and TyG-BMI [14]. While studies have established a connection between TyG levels and CVD risk [15,16], further inquiry into other IR indicators is warranted.

Sarcopenia, marked by an age-related decline in muscle mass and strength, threatens the quality of life for older individuals by increasing the risk of falls, disability, and mortality. Recent studies highlight the link between sarcopenia and CVD, with conditions such as CHD [17,18], atrial fibrillation, heart failure (HF) [19,20], and stroke [21] being associated. Chin et al [22] identified a higher CVD risk in older adults with sarcopenia (odds ratio [OR] 1.768, 95% CI 1.075‐2.909) among 1578 participants aged 65 and older using the Korean population data. A meta-analysis of 38 studies found that sarcopenia prevalence in patients with CVD is 35%, double that of the general population’s 13%, with the highest risks observed in acute decompensated HF (61%) and chronic HF (32%) [8]. Fukuda et al [23] also found a significant relationship between sarcopenic obesity and CVD events, including unstable angina and stroke, in individuals with T2D. There is evidence that sarcopenia is linked to IR, with obesity, inflammation, and hormonal changes possibly explaining their interconnection. IR may disrupt muscle protein balance, leading to decreased muscle mass and functionality, while sarcopenia can impair glucose metabolism [24]. The coexistence of IR and sarcopenia may worsen physical decline through interconnected pathways, increasing CVD risk, particularly in middle-aged and older adults facing additional risk factors. Understanding the combined effects of IR and sarcopenia on CVD risk in older populations is critical for developing targeted prevention and intervention strategies, which could lead to improved health outcomes tailored for this demographic.

## Methods

### Study Design and Participants

This study used a longitudinal cohort design to examine the combined effects of IR and sarcopenia on the incidence of CVD in a middle- and older-aged population. Participants were selected from the China Health and Retirement Longitudinal Study (CHARLS), which spanned 150 counties and 450 communities across 28 provinces between 2011 and 2012. CHARLS has conducted 5 rounds of data collection, including the initial survey and 4 annual follow-ups until 2020 [25].

**Figure 1.**
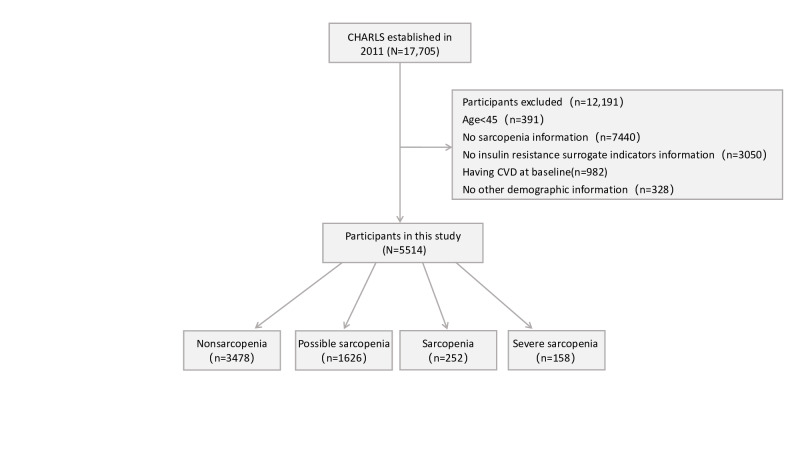
Flowchart for participants included in the study. CHARLS: China Health and Retirement Longitudinal Study; CVD: cardiovascular disease.

For this study, participants aged over 45 without prior CVD at baseline were included, drawn from the CHARLS data collected between 2011 and 2020. Exclusions were made for individuals with incomplete follow-up data, missing baseline sarcopenia status, absent blood samples, or insufficient demographic information. Ultimately, 5514 participants without self-reported CVD at baseline were included, with detailed selection criteria shown in Figure 1.

### Assessment of Sarcopenia Status

Sarcopenia was defined according to the 2019 Asian Working Group for Sarcopenia criteria [26]. Handgrip strength was measured with the Yuejian WL-1000 dynamometer (Nantong Yuejian Physical Measurement Instrument Co. Ltd), with 2 measurements taken for both hands and the maximum value recorded. Diagnostic thresholds were set at <28 kg for men and <18 kg for women. Muscle mass was estimated using the appendicular skeletal muscle mass formula, which considers weight, height, sex, and age, showing concordance with dual-energy X-ray absorptiometry [27]. Participants were classified as having low muscle mass if height-adjusted appendicular skeletal muscle mass fell below 20% of the population, with criteria of <4.90 kg/m² for women and <6.79 kg/m² for men. Low physical performance was defined as a walking speed <1 m/s or a completion time of the 5-time chair stand test exceeding 12 seconds. Sarcopenia was diagnosed when low muscle mass coexisted with either low physical performance or low muscle strength, while those with only one of these indicators were classified as having possible sarcopenia [28-30]. Participants were categorized into 4 groups: nonsarcopenia (n=3478), possible sarcopenia (n=1626), sarcopenia (n=252), and severe sarcopenia (n=158).

### Assessment of CVD Events

The study’s primary outcome was the incidence of CVD, encompassing heart disease and stroke. The time to event was calculated from the baseline assessment to the first recorded CVD event, censoring, or study end, whichever came first. CVD events were identified through self-reported physician diagnoses using specific questions from the CHARLS questionnaire regarding heart disease and stroke diagnoses, such as “Have you been told by a doctor that you have been diagnosed with a heart attack, angina, CHD, HF, stroke, or other heart problems?” This method aligns with previous research [31,32], ensuring consistency and accuracy in CVD identification.

### Calculation of IR Surrogate Indicators

Alongside CVD assessment, surrogate indicators of IR were evaluated. Fasting plasma glucose (FPG), triglycerides, and high-density lipoprotein cholesterol (HDL-C) levels were measured through venous blood samples taken after an overnight fast. Participants’ height, weight, and waist circumference (WC) were measured using standard equipment. In total, 6 surrogate indicators of IR were computed based on validated equations from prior literature [33-36]:

1. TyG=Ln [(TG × FPG) / 2]
2. TyG-BMI=TyG × BMI (BMI=weight/ height², (kg/m²))
3. TyG-WC=TyG × WC
4. TyG-WHtR (waist-to-height ratio)=TyG × WHtR (WHtR=WC (cm) / height (cm))
5. TG/HDL-C=TG/HDL-C
6. METS-IR (metabolic score for insulin resistance)=Ln [(2× FPG)+ TG] × BMI/Ln (HDL-C)

### Assessment of Covariates

Baseline data on participants were collected by trained professionals, covering demographic information, health behaviors, chronic disease history, medication use, and blood test results. Demographics included age, sex, and education level. Health characteristics, such as height, weight, BMI, WC, and WHtR, were assessed, along with participants’ smoking and drinking habits. Chronic diseases and medication use were based on self-reported physician diagnoses, consistent with methodologies for evaluating CVD events in the CHARLS database. No biochemical indicators were used to define these conditions. Data collection adhered to rigorous scientific and ethical standards to ensure study integrity and participant privacy.

### Statistical Analysis

In this study, we first assessed the distributional characteristics of continuous variables. For skewed continuous variables, data were reported as median (IQR), while normally distributed variables were represented as mean (SD). Categorical variables were expressed as counts and percentages. Participants were classified into four groups based on sarcopenia status and IR surrogate index levels at baseline: (1) nonsarcopenia with low-IR levels, (2) high-IR levels only, (3) possible sarcopenia, sarcopenia, and severe sarcopenia only, and (4) coexistence of sarcopenia and high-IR levels. Follow-up time was calculated from the date of the baseline interview (2011‐2012) to the date of the first CVD event, the date of the last follow-up interview (up to 2020), or the date of censoring, whichever occurred first. The median follow-up time for the cohort was 9 years.

The Chi-square test was used for categorical variables, while the Mann-Whitney *U* test was applied to compare continuous variables across groups. We used the Cox proportional hazards model to assess the association between baseline sarcopenic status, IR levels, and incident CVD, calculating hazard ratios (HRs) with 95% CI values. Follow-up time was defined as the interval from the last interview to either the date of CVD diagnosis or the last interview date (March 2019).

Overall, 3 stepwise-adjusted Cox regression models were constructed. Model 1 was unadjusted; Model 2 was adjusted for age and sex; and Model 3 included additional adjustments for smoking status (current and former), alcohol consumption, obesity, hypertension, diabetes, dyslipidemia, and medications for hypertension, diabetes, and dyslipidemia. Variables significant in univariate analysis (*P*<.05) and clinically relevant were included in the multivariate analysis.

Additionally, we performed comprehensive interaction analyses between IR and sarcopenia. Primary analyses focused on possible sarcopenia based on its clinical relevance as an early intervention window and adequate sample size for robust estimation. Secondary exploratory analyses examined interactions with other sarcopenia severity levels (sarcopenia and severe sarcopenia). For multiplicative interaction assessment, product terms (eg, high-IR status × possible sarcopenia status) were included in Cox proportional hazards models. Additive interaction was quantified using the relative excess risk due to interaction (RERI), attributable proportion (AP), and synergy index (SI), which were calculated from HRs derived from Cox models that included a 4-category exposure variable (nonsarcopenia and low-IR, high-IR only, possible sarcopenia only, and both conditions).

To establish clinically applicable cutoff values for identifying high-risk individuals, we performed receiver operating characteristic (ROC) curve analysis. The optimal cutoff points for each IR index and sarcopenia-related measure were determined by maximizing the Youden index (J=sensitivity + specificity – 1), which provides the optimal balance between sensitivity and specificity for predicting incident CVD. This analysis was conducted using the pROC package in R, and the resulting cutoff values are presented in Table S6 in Multimedia Appendix 1. All statistical analyses were conducted using R statistical software (version 4.3.2; R Core Team), with a *P* value of less than .05 considered statistically significant.

### Ethical Considerations

The CHARLS study protocol was approved by the Biomedical Ethics Committee of Peking University (IRB 00001052‐11015). All participants provided written informed consent at the time of the original data collection. This study involved a secondary analysis of publicly available, deidentified CHARLS data. No direct contact with participants occurred, and no individual-level identifiable information was accessed. Therefore, additional ethics approval and informed consent were not required, as the original consent and institutional review board approval covered secondary use of the dataset. All data used in this study were fully anonymized to ensure participant privacy and confidentiality. Participants in the original CHARLS survey received compensation according to the study’s standard protocol.

## Results

### Overview

The baseline characteristics of the participants stratified by the presence of CVD are presented in Table 1. A total of 5514 participants were included in the analysis, with 4529 classified as non-CVD and 985 as CVD cases. The median age of participants was 62 years, and 2157 (39%) were female. Among those with CVD, 550 (56%) were female. During a median follow-up of 9 years, 985 new cases of CVD were recorded, constituting 17.86% of the total. All 6 IR indicators (TyG, TyG-BMI, TyG-WC, TyG-WHtR, TG/HDL-C, and METS-IR) exhibited significantly elevated levels in patients with new-onset CVD compared to the control group without CVD (*P*<.001). The prevalence rates of sarcopenia by group were 63% for nonsarcopenia, 29% for possible sarcopenia, 4.6% for sarcopenia, and 2.9% for severe sarcopenia.

**Table 1.** Baseline patient characteristics grouped by outcomes.

Variables	All (N=5514)	Non-CVD^a^ (n=4529)	CVD (n=985)	*P* value
Age (years)	62 (59-68)	62 (59-68)	62 (59-67)	.40
Sex, n (%)				<.001
Female	2707 (49)	2157 (48)	550 (56)	
Male	2807 (51)	2372 (52)	435 (44)	
Education, n (%)				.60
College or university	62 (1.1)	48 (1.1)	14 (1.4)	
Illiterate	1880 (34)	1537 (34)	343 (35)	
Primary school	2530 (46)	2078 (46)	452 (46)	
Secondary or high school	1032 (19)	858 (19)	174 (18)	
Systolic blood pressure (mm Hg), median (IQR)	130 (117-144)	129 (116-143)	134 (121-149)	<.001
Diastolic blood pressure (mm Hg), median (IQR)	74 (67-81)	74 (67-81)	76 (68-84)	<.001
HbA_1c_^b^, median (IQR)	5.20 (4.90-5.40)	5.20 (4.90-5.40)	5.20 (4.90-5.50)	.11
TG^c^ (mg/dL), median (IQR)	103 (74-148)	100 (73-146)	115 (81-160)	<.001
TC^d^ (mg/dL), median (IQR)	192 (168-217)	191 (167-216)	196 (173-222)	<.001
HDL-C^e^ (mg/dL), median (IQR)	50 (41-61)	51 (41-61)	48 (40-60)	.002
LDL-C^f^ (mg/dL), median (IQR)	116 (95-139)	115 (94-138)	119 (99-143)	<.001
WC^g^ (cm), median (IQR)	85 (78-92)	85 (78-92)	87 (79-95)	<.001
Hypertension, n (%)	2395 (44)	1845 (41)	550 (56)	<.001
Diabetes, n (%)	415 (9.8)	305 (8.9)	110 (14)	<.001
Dyslipidemia, n (%)	1009 (25)	789 (24)	220 (29)	.002
Smoking, n (%)				<.001
Ex-smoker	466 (8.6)	378 (8.5)	88 (9.1)	
Nonsmoker	3197 (59)	2577 (58)	620 (64)	
Smoker	1735 (32)	1481 (33)	254 (26)	
Drinking, n (%)	1895 (34)	1584 (35)	311 (32)	.046
TyG^h^, median (IQR)	8.59 (8.23-9.02)	8.56 (8.21-9)	8.71 (8.32-9.16)	<.001
Obesity, n (%)	2113 (38)	1660 (37)	453 (46)	<.001
WHtR^i^, median (IQR)	0.54 (0.49-0.59)	0.54 (0.49-0.59)	0.55 (0.50-0.61)	<.001
TyG-BMI , median (IQR)	198 (173-226)	196 (172-223)	207 (182-236)	<.001
TyG-WC, median (IQR)	731 (650-815)	725 (646-806)	754 (673-851)	<.001
TyG-WHtR , median (IQR)	4.66 (4.14-5.25)	4.62 (4.10-5.20)	4.85 (4.33-5.45)	<.001
TG/HDLC, median (IQR)	2.05 (1.28-3.40)	1.97 (1.26-3.36)	2.31 (1.46-3.70)	<.001
METS-IR^j^, median (IQR)	34 (29-40)	34 (29-39)	36 (31-42)	<.001
Sarcopenia state, n (%)				<.001
Nonsarcopenia	3478 (63)	2912 (64)	566 (57)	
Possible sarcopenia	1626 (29)	1273 (28)	353 (36)	
Sarcopenia	252 (4.6)	211 (4.7)	41 (4.2)	
Severe sarcopenia	158 (2.9)	133 (2.9)	25 (2.5)	

a CVD: cardiovascular disease.

b HbA_1c_: glycosylated hemoglobin.

c TG: triglyceride.

d TC: total cholesterol.

e HDL-C: high-density lipoprotein cholesterol.

f LDL-C: low-density lipoprotein cholesterol.

g WC: waist circumference.

h TyG: triglyceride glucose.

i WHtR: waist-to-height ratio.

j METS-IR: metabolic score for insulin resistance.

Compared to participants without a history of CVD, those diagnosed with CVD had higher prevalence rates of obesity, increased WC, and higher levels of triglyceride, total cholesterol, and low-density lipoprotein cholesterol. Additionally, the prevalence of hypertension, diabetes, and dyslipidemia was significantly greater in the CVD group (*P*<.001). A detailed account of these demographic and clinical characteristics is illustrated in Table 1. Due to the results of the normality test indicating non-normal distribution, continuous variables are expressed as median (IQR) in Table 1, providing a more accurate representation of central tendency and variability.

### Cox Regression Analysis of Baseline IR and Sarcopenia on Follow-Up CVD

Table 2 and Table S1 in Multimedia Appendix 1 detailed the association between the 6 IR surrogate indicators (TyG, TyG-BMI, TyG-WC, TyG-WHtR, TG/HDL-C, and METS-IR) and the risk of CVD, alongside the status of sarcopenia. In multivariate analysis, a significant association was found between TyG (per SD increment) and the risk of incident CVD (HR 1.176, 95% CI 1.030-1.343). Other IR surrogates, such as TyG-BMI (HR 1.004, 95% CI 1.001-1.007) and TyG-WC (HR 1.001, 95% CI 1.000-1.002), also demonstrated statistically significant relationships with increased CVD risk. Conversely, TG/HDL-C showed an attenuated result, with no significant relationship (HR 1.06, 95% CI 0.99-1.14). In mutual adjustment models, TyG, TyG-BMI, and TyG-WC remained statistically significant.

When sarcopenia status was categorized into 4 types, possible sarcopenia and severe sarcopenia were associated with an increased risk of CVD compared to the nonsarcopenia population, after adjusting for other confounders (HR 1.320, 95% CI 1.125-1.549) for possible sarcopenia and 1.709 (1.075-1.763) for severe sarcopenia. However, the risk of CVD associated with the sarcopenia group did not reach statistical significance (HR 1.181, 95% CI 0.791-1.763). Furthermore, significant associations were observed between possible sarcopenia and severe sarcopenia with the risk of incident CVD in multivariable analyses, even after adjustments for IR surrogate indicators. These findings indicate that both IR and sarcopenia play critical roles in predicting CVD risk.

**Table 2.** Relationship of insulin resistance surrogate indicators or sarcopenia status with cardiovascular disease risk.

Characteristics	Crude model, HR^a^ (95% CI)	*P* value	Adjusted model^b^, HR (95% CI)	*P* value	*P* for interaction^c^
IR^d^ surrogate^e^ indicators					
TyG^f^ (per SD)	1.312 (1.186‐1.451)	<.001	1.176 (1.030‐1.343)	.02	.003
TyG-BMI (per SD)	1.006 (1.004‐1.008)	<.001	1.004 (1.001‐1.007)	.02	.01
TyG-WC^g^ (per SD)	1.002 (1.001‐1.002)	<.001	1.001 (1‐1.002)	.02	.06
TyG-WHtR^h^ (per SD)	1.275 (1.174‐1.385)	<.001	1.096 (0.985‐1.219)	.09	.04
TG^i^/HDL-C^j^ (per SD)	1.035 (1.013‐1.057)	<.001	1.007 (0.943‐1.082)	.26	<.001
METS-IR^k^ (per SD)	1.024 (1.015‐1.033)	<.001	1.011 (0.980‐1.035)	.12	.001
Sarcopenia status					
Nonsarcopenia	1 (Reference)	—^l^	1 (Reference)	—	—
Possible sarcopenia	1.462 (1.280‐1.670)	<.001	1.320 (1.125‐1.549)	.001	—
Sarcopenia	1.171 (0.853‐1.608)	.33	1.181 (0.791‐1.763)	.42	—
Severe sarcopenia	1.311 (0.878‐1.957)	.19	1.709 (1.075‐2.715)	.02	—

a HR: hazard ratio.

b Adjusted model: adjusted for age, sex, smoking, drinking, hypertension, diabetes, dyslipidemia, hypertension medications, diabetes medications, and dyslipidemia medications.

c An interaction between IR surrogate indicators and sarcopenia status.

d IR: insulin resistance.

e IR surrogate indicators are analyzed as a continuous variable in the table.

f TyG: triglyceride glucose.

g WC: waist circumference.

h WHtR: waist-to-height ratio.

i TG: triglyceride.

j HDL-C: high-density lipoprotein cholesterol.

k METS-IR: metabolic score for insulin resistance.

l Not available.

### The Combination of IR and Sarcopenia Increased the Risk

Table 3 illustrates the crude and multivariable adjusted associations between the primary exposures and CVD risk. To illustrate, consider the combination of TyG and sarcopenia status, the incident CVD rates showed the following HRs in the unadjusted model: 1.44 (1.19-1.75) for nonsarcopenia and high TyG, 1.43 (1.12-1.82) for possible sarcopenia and low TyG, 2.01 (1.63-2.48) for possible sarcopenia and high TyG, and 1.86 (1.08-3.20) for severe sarcopenia and low TyG groups, relative to nonsarcopenia and low TyG. These associations remained statistically significant in Model 2, adjusted for age and sex, except for the severe sarcopenia/low TyG group.

After adjusting for potential confounders, including smoking and drinking habits, and chronic disease history (Model 3), we found that possible sarcopenia and high TyG is associated with a 1.53-fold higher hazard of experiencing CVD (HR 1.53, 95% CI 1.20-1.96), while sarcopenia and low TyG was associated with a 1.37-fold higher hazard (HR 1.37, 95% CI 1.07-1.76). The combination of other IR surrogates with sarcopenia also demonstrated similar patterns, with participants experiencing heightened CVD risk across various combinations of IR indicators and sarcopenia statuses (eg, HR 1.32, 95% CI 1.02‐1.71) for possible sarcopenia with low TyG, 1.79 (1.30‐2.46) for possible sarcopenia with high METS-IR). These findings emphasize the significance of considering both IR and sarcopenia in assessing CVD risk.

**Table 3.** Association of sarcopenia status and insulin resistance surrogate indicators with new onset of cardiovascular disease.

Groups^a^	Model 1^b^, HR^c^ (95% CI)	Model 2^d^, HR (95% CI)	Model 3^e^, HR (95% CI)
Nonsarcopenia and low TyG^f^	1 (Reference)	1 (Reference)	1 (Reference)
Nonsarcopenia and high TyG	1.44 (1.19‐1.75)^g^	1.41 (1.17‐1.71)^g^	1.18 (0.96‐1.46)
Possible sarcopenia and low TyG	1.43 (1.12‐1.82)^h^	1.37 (1.07‐1.76)^i^	1.32 (1.02‐1.71)^i^
Possible sarcopenia and high TyG	2.01 (1.63‐2.48)^g^	1.83 (1.47‐2.28)^g^	1.53 (1.20‐1.96)^g^
Severe sarcopenia and low TyG	1.86 (1.08‐3.20)^i^	1.35 (0.74‐2.48)	1.68 (0.89‐3.19)
Nonsarcopenia and low TyG-BMI	1 (Reference)	1 (Reference)	1 (Reference)
Nonsarcopenia and high TyG-BMI	1.66 (1.37‐2.02)^g^	1.65 (1.35‐2.01)^g^	1.25 (0.94‐1.67)
Possible sarcopenia and low TyG-BMI	1.58 (1.22‐2.03)^g^	1.50 (1.15‐1.95)^h^	1.47 (1.12‐1.92)^h^
Possible sarcopenia and high TyG-BMI	2.18 (1.75‐2.70)^g^	2.02 (1.61‐2.52)^g^	1.69 (1.20‐2.39)^h^
Sarcopenia and low TyG-BMI	1.50 (1.03‐2.20)^i^	1.21 (0.80‐1.83)	1.13 (0.72‐1.76)
Severe sarcopenia and low TyG-BMI	2.12 (1.35‐3.31)^g^	1.52 (0.90‐2.56)	1.50 (0.89‐2.54)
Nonsarcopenia and low TyG-WC^j^	1 (Reference)	1 (Reference)	1 (Reference)
Nonsarcopenia and high TyG-WC	1.64 (1.35‐1.99)^g^	1.62 (1.33‐1.96)^g^	1.26 (0.98‐1.61)
Possible sarcopenia and low TyG-WC	1.78 (1.40‐2.28)^g^	1.71 (1.33‐2.20)^g^	1.67 (1.29‐2.17)^g^
Possible sarcopenia and high TyG-WC	1.95 (1.57‐2.41)^g^	1.80 (1.44‐2.25)^g^	1.30 (0.97‐1.74)
Severe sarcopenia and low TyG-WC	2.02 (1.25‐3.24)^h^	1.38 (0.79‐2.39)	1.52 (0.86‐2.68)
Nonsarcopenia and low TyG-WHtR^k^	1 (Reference)	1 (Reference)	1 (Reference)
Nonsarcopenia and high TyG-WHtR	1.64 (1.35‐1.99)^g^	1.59 (1.31‐1.94)^g^	1.15 (0.89‐1.48)
Possible sarcopenia and low TyG-WHtR	1.80 (1.40‐2.30)^g^	1.74 (1.36‐2.24)^g^	1.71 (1.32‐2.21)^g^
Possible sarcopenia and high TyG-WHtR	1.92 (1.56‐2.37)^g^	1.90 (1.51‐2.39)^g^	1.29 (0.97‐1.73)
Sarcopenia and low TyG-WHtR	1.56 (1.06‐2.30)^i^	1.29 (0.84‐1.97)	1.27 (0.80‐2.01)
Severe sarcopenia and low TyG-WHtR	1.88 (1.11‐3.19)^i^	1.40 (0.77‐2.56)	1.62 (0.88‐2.96)
Severe sarcopenia and high TyG-WHtR	2.64 (1.24‐5.62)^i^	2.25 (1.00‐5.07)	1.64 (0.67‐4.04)
Nonsarcopenia and low TG^l^/HDL-C^m^	1 (Reference)	1 (Reference)	1 (Reference)
Nonsarcopenia and high TG/HDL-C	1.46 (1.21‐1.76)^g^	1.45 (1.20‐1.75)^g^	1.27 (1.03‐1.56)^i^
Possible sarcopenia and low TG/HDL-C	1.36 (1.06‐1.75)^i^	1.31 (1.01‐1.69)^g^	1.28 (0.99‐1.67)
Possible sarcopenia and high TG/HDL-C	2.09 (1.70‐2.57)^g^	1.92 (1.55‐2.38)^g^	1.67 (1.32‐2.13)^g^
Sarcopenia and high TG/HDL-C	1.92 (1.04‐3.53)^i^	1.47 (0.76‐2.82)	1.73 (0.86‐3.48)
Severe sarcopenia and low TG/HDL-C	1.76 (1.02‐3.03)^i^	1.27 (0.69‐2.34)	1.66 (0.89‐3.08)
Severe sarcopenia and high TG/HDL-C	2.30 (1.13‐4.66)^i^	1.70 (0.79‐3.63)	1.68 (0.73‐3.85)
Nonsarcopenia and low METS-IR^n^	1 (Reference)	1 (Reference)	1 (Reference)
Nonsarcopenia and high METS-IR	1.61 (1.33‐1.96)^g^	1.59 (1.31‐1.94)^g^	1.23 (0.94‐1.61)
Possible sarcopenia and low METS-IR	1.53 (1.19‐1.97)^h^	1.43 (1.10‐1.86)^h^	1.38 (1.05‐1.81)^i^
Possible sarcopenia and high METS-IR	2.15 (1.74‐2.66)^g^	2.00 (1.60‐2.49)^g^	1.79 (1.30‐2.46)^g^
Sarcopenia and high METS-IR	2.06 (1.12‐3.79)^i^	1.46 (0.76‐2.82)	1.52 (0.76‐3.05)
Severe sarcopenia and low METS-IR	2.10 (1.34‐3.27)^h^	1.43 (0.85‐2.41)	1.49 (0.88‐2.52)

a IR surrogate category is divided into high-value and low-value groups based on the median.

b Hazard ratios for combinations of sarcopenia and IR that were statistically significant (*P*<.05) in the unadjusted Model 1 combinations not significant in Model 1 are not shown. Model 1: unadjusted.

c HR: hazard ratio.

d Model 2: adjusted for age and sex.

e Model 3: adjusted as model 2 with further adjustment for smoking, drinking, hypertension, diabetes, dyslipidemia, hypertension medications, diabetes medications, and dyslipidemia medications.

f TyG: triglyceride glucose.

g *P*<.001.

h *P*<.01.

i *P*<.05.

j WC: waist circumference.

k WHtR: waist-to-height ratio.

l TG: triglyceride.

m HDL-C: high-density lipoprotein cholesterol.

n METS-IR: metabolic score for insulin resistance.

### Interaction Analyses Between IR and Sarcopenia

To quantitatively characterize the joint effects of IR and sarcopenia on CVD risk, we conducted both multiplicative and additive interaction analyses using median-based cutoffs for IR indices.

Multiplicative interaction analysis revealed significant interactions for 2 indices: TyG-WC (HR 0.728, 95% CI 0.533‐0.995; *P*=.046) and TyG-WHtR (HR 0.715, 95% CI 0.523‐0.979; *P*=.04). For other IR indices, multiplicative interaction terms were not statistically significant (all *P*>.05). Additive interaction analysis, assessed using RERI, AP, and S, demonstrated distinct patterns: TG/HDL-C ratio showed clear synergistic effects (RERI=0.139, AP=0.083, SI=1.263); METS-IR demonstrated synergistic effects (RERI=0.039, AP=0.025, SI=1.075); TyG exhibited mild synergistic effects (RERI=0.045, AP=0.029, SI=1.089); Other indices (TyG-BMI, TyG-WC, and TyG-WHtR) showed antagonistic effects (negative RERI values). Complete results of both multiplicative and additive interaction analyses are presented in Table S2-5, in Multimedia Appendix 1.

### Subgroup Analysis

**Figure 2.**
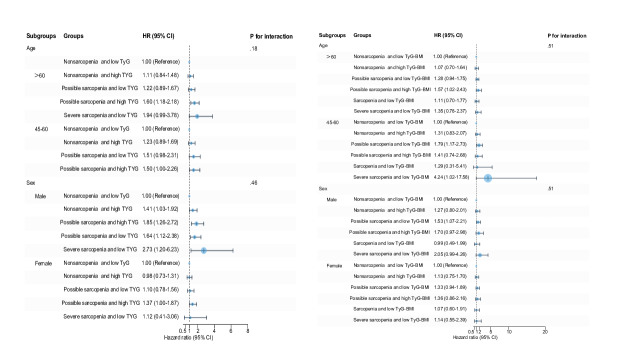
Subgroup analysis of insulin resistance surrogate indicators and sarcopenia status with cardiovascular disease risk. Hazard ratios were adjusted for age, sex, smoking, drinking, hypertension, diabetes, dyslipidemia, hypertension medications, diabetes medications, and dyslipidemia medications. HR: hazard ratio; TyG: triglyceride glucose.

Figures2^4^ display the significant interplay between IR levels and sarcopenia status on CVD risk across different age and sex subgroups. Stratified Cox regression analyses, referenced against the nonsarcopenia with low IR (non_sp.IR_L) group, revealed distinct age- and sex-specific associations between sarcopenia severity and IR profiles. Notably, possible sarcopenia combined with high IR consistently represented the highest risk profile, particularly in younger adults (45‐60 y). Even in low-IR states, those with possible sarcopenia and low TyG-WHtR independently increased their risk by 2.5-fold (HR 2.51, 95% CI 1.69‐3.73), indicating potential sarcopenia-driven pathways beyond mere metabolic dysregulation. In male participants, possible sarcopenia combined with high IR, particularly lipid-centric markers such as elevated TG/HDL-C, conferred the greatest risk. In female participants, combinations of possible sarcopenia with high-IR indices linked to central adiposity (eg, TyG-WC) significantly elevated risk, with even low-IR subgroups exhibiting independent risks driven by sarcopenia.

Due to small subgroup sizes for sarcopenia (n=252) and severe sarcopenia (n=158), analyses were limited in precision for extreme risk estimates. Models adjusted for age, sex, smoking status, drinking, obesity, hypertension, diabetes, dyslipidemia, and medications confirmed the robustness of findings, underscoring the dual contribution of sarcopenia and IR to CVD pathogenesis. These results advocate for integrated clinical strategies targeting muscle health and metabolic dysregulation, particularly in high-risk subgroups characterized by possible sarcopenia and elevated IR.

**Figure 3.**
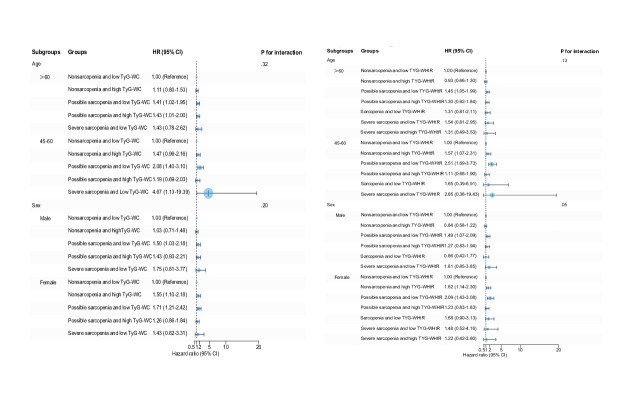
Subgroup analysis of insulin resistance surrogate indicators and sarcopenia status with cardiovascular disease risk. Hazard ratios were adjusted for age, sex, smoking, drinking, hypertension, diabetes, dyslipidemia, hypertension medications, diabetes medications, and dyslipidemia medications. HR: hazard ratio; TyG: triglyceride glucose; WC: waist circumference; WHtR: waist-to-height ratio.

**Figure 4.**
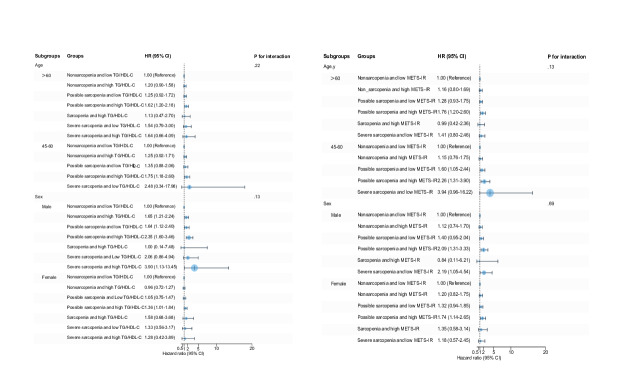
Subgroup analysis of insulin resistance surrogate indicators and sarcopenia status with cardiovascular disease risk. Hazard ratios were adjusted for age, sex, smoking, drinking, hypertension, diabetes, dyslipidemia, hypertension medications, diabetes medications, and dyslipidemia medications. HR: hazard ratio; METS-IR: metabolic score for insulin resistance; TG/HDL-C: triglyceride/high-density lipoprotein cholesterol; TyG: triglyceride glucose.

### Clinically Applicable Cutoff Values

To facilitate the identification of high-risk individuals in clinical practice, we determined optimal cutoff values for all IR indices and sarcopenia measures using ROC analysis. Key cutoff values include: TG/HDL-C ratio 2.09 (sensitivity 58.8% and specificity 53.4%), METS-IR 34.26 (sensitivity 58.2% and specificity 54%), TyG-BMI 192.47 (sensitivity 65.9% and specificity 46.4%), handgrip strength 26.85 kg, and chair stand test time 9.41 seconds. The complete results, including cutoff values, sensitivity, specificity, and area under the curve, are presented in Table S6 in Multimedia Appendix 1.

## Discussion

### Principal Findings

In this longitudinal study, we observed a synergistic interplay between IR and sarcopenia regarding the risk of CVD among middle-aged and older individuals. Notably, the combination of high IR and possible sarcopenia exhibited the highest cardiovascular risk, particularly in those with elevated METS-IR levels, when compared to individuals in the nonsarcopenia and low METS-IR group. The prospective design ensures that assessments of sarcopenia and IR at baseline preceded CVD onset during follow-up, strengthening the inference of a causal pathway and reducing concerns of reverse causality. This research systematically correlates multidimensional IR indices with sarcopenia staging within a large national cohort, offering new insights into the confluence of IR and sarcopenia’s impact on CVD risk.

Previous studies have demonstrated the significant positive association between the METS-IR index and CVD risk. Duan et al [37] found that METS-IR exhibited a stronger association with cardiovascular mortality compared to the TyG index, TG/HDL-C, and Homeostatic Model Assessment for Insulin Resistance, particularly in individuals under 65 years old. Another prospective cohort study comprising 47,270 participants from the Kailuan Study also indicated that elevated METS-IR levels increased CVD risk. The TyG index and TG/HDL-C ratio are increasingly recognized as surrogate markers of IR, confirming their relevance in CVD risk assessment. Previous research supports this association; for example, Salazar et al [38] conducted a 9-year prospective study involving 796 participants, finding a higher TG/HDL-C ratio as a predictor of incident CVD events. Similarly, Che et al [39] presented evidence of a significant correlation between elevated TyG and TG/HDL-C indices and increased CVD risks using the UK Biobank population. Further supporting these findings, a cross-sectional study involving 13,706 participants indicated that the TyG index independently predicts 10-year CVD risk, outperforming traditional IR measures such as Homeostatic Model Assessment for Insulin Resistance [14]. Additionally, Hong et al [40] also reported a robust link between elevated TyG indices and atherosclerotic CVD risk in older adults, highlighting the necessity of identifying optimal TyG levels for early intervention. In a retrospective cohort analysis involving 14,653 adults from the NHANES database (2001‐2018), Li et al [41] conducted a longitudinal study based on the CHARLS database (2011‐2015) and established a significant positive association between cumulative average TyG-BMI and incident CVD. Moreover, another longitudinal study from the CHARLS database involving 7115 participants demonstrated that TyG, TyG-BMI, TyG-WC, and TyG-WHtR were all significantly associated with new-onset CVDs. Notably, the TyG index outperformed the modified TyG indices in identifying CVD risk over a 7-year follow-up period [42]. In this study, we found that TyG, TyG-BMI, and TyG-WC functioned as robust predictors of CVD risk, independent of traditional confounders. Conversely, the associations of TyG-WHtR, TG/HDL-C, and METS-IR attenuated to nonsignificance after adjusting for factors such as age, sex, smoking, drinking habits, hypertension, diabetes, dyslipidemia, and obesity. These results highlight the critical role of specific IR markers in predicting CVD risk and support the relevance of the TyG index and its derivatives in clinical assessments and interventions aimed at improving cardiovascular health.

Our findings revealed that sarcopenia, alongside IR, serves as a significant predictor of CVD risk. We demonstrated that individuals with possible sarcopenia were at a higher risk for CVD compared to those without sarcopenia. In line with this, Gao et al [29] noted the association between possible sarcopenia and increased CVD risk, while Chai et al [43] large Taiwanese cohort study confirmed that sarcopenia correlated with a higher prevalence of stroke in patients with T2D. Xia et al [18] further showed that sarcopenia independently increased the risks of myocardial infarction and atrial fibrillation in middle-aged and older adults. However, our study presented sarcopenia status in 4 categories, revealing no statistically significant difference concerning CVD risk among sarcopenic groups, possibly attributable to limited sample sizes. Thus, more prospective multicenter studies are warranted for further validation. Given these findings, targeted interventions for patients diagnosed with sarcopenia, especially those with possible sarcopenia, are crucial. These interventions may include physical therapy to enhance muscle strength and function, along with medications to improve muscle health, thereby potentially reducing the incidence of CVD.

The mechanisms underlying this synergistic effect remain unclear; however, previous studies provide valuable insights. Skeletal muscle is pivotal for insulin-mediated glucose disposal postmeal [44,45]. With IR, glucose metabolism in muscle is disrupted, leading to decreased protein synthesis and increased breakdown, contributing to sarcopenia development [45]. Previous studies have found that IR can impair PI3K-Akt signaling, resulting in reduced glucose uptake, suppression of mTOR-mediated protein synthesis, and activation of the ubiquitin-proteasome system responsible for protein degradation [46]. Aging exacerbates these issues, as adipose tissue inflammation can lead to fat accumulation in the intra-abdominal region (visceral fat). Fatty infiltration in skeletal muscle leads to diminished strength and functionality [47]. Increased production of adipokines due to visceral fat promotes muscle loss through pro-inflammatory cytokines, which counteract anabolic signals from myokines, fostering a chronic inflammatory state that aggravates both IR and muscle degeneration [48,49]. Together, these processes contribute to CVD’s onset and progression through mechanisms involving metabolic disturbances, inflammation, oxidative stress, and vascular impairment [50-52].

In this study, we specifically demonstrated that individuals with both possible sarcopenia and high IR had a substantially enhanced CVD risk from 1.24-1.85 fold compared to the independent effects of these conditions. While prior studies typically approached sarcopenia dichotomously, our stratification by severity revealed a nonlinear relationship: early-stage sarcopenia may represent a critical window for metabolic intervention, while advanced muscle loss risks appear to follow a different risk trajectory. This pattern was reinforced in our subgroup analyses, which showed that the combination of possible sarcopenia and high IR consistently conferred robust and statistically significant risk across demographic strata. Interestingly, we observed numerically elevated, though statistically imprecise, HRs for severe sarcopenia even in subgroups with low-IR levels (Figure 2). This suggests that advanced muscle loss may itself constitute an independent risk factor, potentially operating through pathways distinct from metabolic dysregulation. However, the instability of these estimates, attributable to limited sample sizes in severe sarcopenia subgroups, necessitates cautious interpretation and underscores the need for future studies with larger cohorts to definitively characterize these relationships.

Formal interaction analyses further provided quantitative evidence supporting a synergistic relationship between IR and sarcopenia. Although interaction patterns varied across IR indices, the TG/HDL-C ratio and METS-IR consistently demonstrated synergistic effects on both additive and multiplicative scales. These composite measures, which integrate lipid metabolism, glucose homeostasis, and adiposity, may be particularly sensitive to the pathophysiological interplay between metabolic health and muscle quality. Their consistent performance highlights the biological complexity of IR and its connection to sarcopenia, while reinforcing our primary finding that the combination of possible sarcopenia and high IR confers the greatest CVD risk.

The ROC-derived cutoff values established in this study bridge the gap between epidemiological evidence and clinical practice by providing tangible thresholds for risk identification. Although single indices had modest discrimination (area under the curve 0.50‐0.58), the ROC-derived cutoffs nevertheless provide practical screening thresholds to identify individuals who warrant combined metabolic and muscle health assessment. Particularly noteworthy is the alignment between the cutoff values for TG/HDL-C ratio (2.09) and METS-IR (34.26) and our interaction analyses, which identified these indices as exhibiting the most consistent synergistic effects with possible sarcopenia. These findings support a practical 2-step approach to CVD risk stratification in middle-aged and older adults: (1) initial screening using the established cutoff values to identify individuals with high IR, impaired muscle health, or both; (2) focused intervention for those with both conditions, especially the combination of high TG/HDL-C (≥2.09) or high METS-IR (≥34.26) with possible sarcopenia, as this profile confers the highest CVD risk. The accessibility and calculability of these indices enhance their use in primary care and community settings where complex diagnostic procedures may be unavailable. Furthermore, the early identification of possible sarcopenia, a potentially reversible stage, combined with metabolic assessment, creates a critical window for implementing dual-targeted interventions aimed at preserving muscle mass while improving metabolic health.

Collectively, these findings emphasize the necessity of tailored management strategies addressing both sarcopenia and IR, particularly during the early stages of muscle decline. Proactive intervention during this critical window may improve clinical outcomes and attenuate CVD progression.

While this study elucidates the synergistic effect of sarcopenia staging and IR on CVD risk within a nationally representative older adults cohort, it is subject to certain limitations. First, the reliance on self-reported CVD diagnoses may introduce misclassification bias. We were unable to validate incident CVD outcomes against medical records, administrative claims, or mortality registries. Consequently, outcome misclassification may have occurred. If such misclassification is nondifferential with respect to the exposures studied, it would likely bias HR estimates toward the null, implying that the true associations might be stronger than those observed. However, differential misclassification (eg, systematic underreporting among older, less educated, or rural participants) could bias estimates in either direction, and we were not able to quantify the magnitude of these potential biases in the current dataset. Future studies that link CHARLS participants to hospital records, insurance claims, or death registries, or that apply adjudicated outcome ascertainment, would substantially strengthen the validity of outcome assessment. Second, the wide CI values in severe sarcopenia subgroups due to small sample sizes necessitate cautious interpretation, limiting the generalizability of findings to a Chinese-specific context. Moreover, we were unable to monitor changes in IR levels and sarcopenia status over time, restricting our understanding of their dynamic roles in CVD development. Future studies should incorporate additional potential confounders and explore the long-term effects of changes in IR and sarcopenia status on CVD risk, guiding precision interventions.

### Conclusions

In conclusion, this study has evidenced that IR and sarcopenia, particularly in their early possible stage, interact synergistically to elevate CVD risk in middle-aged and older adults. The findings underscore the urgent need for early identification and intervention strategies, especially for individuals experiencing possible sarcopenia alongside high-IR levels. Such proactive approaches could significantly influence patient outcomes, reducing cardiovascular event incidence and enhancing prognosis and quality of life for middle-aged and older adults. However, further prospective studies are essential to confirm these findings and clarify the mechanisms behind the interaction between IR and sarcopenia. Addressing these gaps will be critical in developing effective clinical strategies to manage these conditions and improve health outcomes in this vulnerable population.

## Supplementary material

10.2196/80115 Multimedia Appendix 1 Analyses of the associations and interactions between insulin resistance surrogate indicators, sarcopenia, and cardiovascular disease risk.

## Abbreviations

AP: attributable proportion
CHARLS: China Health and Retirement Longitudinal Study
CHD: coronary heart disease
CVD: cardiovascular disease
FPG: fasting plasma glucose
HDL-C: high-density lipoprotein cholesterol
HF: heart failure
HR: hazard ratio
IR: insulin resistance
METS-IR: metabolic score for insulin resistance
RERI: relative excess risk due to interaction
ROC: receiver operating characteristic
SI: synergy index
T2D: type 2 diabetes
TyG: triglyceride glucose
WC: waist circumference
WHtR: waist-to-height ratio

## References

[1] The Lancet Public Health (2017). Ageing: a 21st century public health challenge?. Lancet Public Health.

[2] Global Burden of Cardiovascular Diseases and Risks 2023 Collaborators (2025). Global, regional, and national burden of cardiovascular diseases and risk factors in 204 countries and territories, 1990-2023. J Am Coll Cardiol.

[3] National Center For Cardiovascular Diseases The Writing Committee Of The Report On Cardiovascular Health And Diseases In China 1 (2024). Report on cardiovascular health and diseases in China 2023: an updated summary. Biomed Environ Sci.

[4] Zhou M, Zhao G, Zeng Y, Zhu J, Cheng F, Liang W (2022). Aging and cardiovascular disease: current status and challenges. Rev Cardiovasc Med.

[5] GBD 2016 Causes of Death Collaborators (2017). Global, regional, and national age-sex specific mortality for 264 causes of death, 1980-2016: a systematic analysis for the Global Burden of Disease Study 2016. Lancet.

[6] Dunbar SB, Khavjou OA, Bakas T (2018). Projected costs of informal caregiving for cardiovascular disease: 2015 to 2035: a policy statement from the American Heart Association. Circulation.

[7] Corbett S, Courtiol A, Lummaa V, Moorad J, Stearns S (2018). The transition to modernity and chronic disease: mismatch and natural selection. Nat Rev Genet.

[8] Zuo X, Li X, Tang K (2023). Sarcopenia and cardiovascular diseases: a systematic review and meta-analysis. J Cachexia Sarcopenia Muscle.

[9] Castro L, Brant L, Diniz M de F (2023). Association of hypertension and insulin resistance in individuals free of diabetes in the ELSA-Brasil cohort. Sci Rep.

[10] Pyörälä M, Miettinen H, Halonen P, Laakso M, Pyörälä K (2000). Insulin resistance syndrome predicts the risk of coronary heart disease and stroke in healthy middle-aged men: the 22-year follow-up results of the Helsinki Policemen Study. Arterioscler Thromb Vasc Biol.

[11] Hill MA, Yang Y, Zhang L (2021). Insulin resistance, cardiovascular stiffening and cardiovascular disease. Metab Clin Exp.

[12] Shulman GI, Rothman DL, Jue T, Stein P, DeFronzo RA, Shulman RG (1990). Quantitation of muscle glycogen synthesis in normal subjects and subjects with non-insulin-dependent diabetes by 13C nuclear magnetic resonance spectroscopy. N Engl J Med.

[13] Guerrero-Romero F, Simental-Mendía LE, González-Ortiz M (2010). The product of triglycerides and glucose, a simple measure of insulin sensitivity. Comparison with the euglycemic-hyperinsulinemic clamp. J Clin Endocrinol Metab.

[14] Guo W, Zhu W, Wu J (2021). Triglyceride glucose index is associated with arterial stiffness and 10-year cardiovascular disease risk in a Chinese population. Front Cardiovasc Med.

[15] Lopez-Jaramillo P, Gomez-Arbelaez D, Martinez-Bello D (2023). Association of the triglyceride glucose index as a measure of insulin resistance with mortality and cardiovascular disease in populations from five continents (PURE study): a prospective cohort study. Lancet Healthy Longev.

[16] Zhang N, Chi X, Zhou Z (2023). Triglyceride-glucose index is associated with a higher risk of stroke in a hypertensive population. Cardiovasc Diabetol.

[17] Santana N de M, Mendes RML, Silva N da, Pinho CPS (2019). Sarcopenia and sarcopenic obesity as prognostic predictors in hospitalized elderly patients with acute myocardial infarction [Article in English, Portuguese]. Einstein (Sao Paulo).

[18] Xia MF, Chen LY, Wu L (2021). Sarcopenia, sarcopenic overweight/obesity and risk of cardiovascular disease and cardiac arrhythmia: a cross-sectional study. Clin Nutr.

[19] Curcio F, Testa G, Liguori I (2020). Sarcopenia and heart failure. Nutrients.

[20] Suzuki T, Palus S, Springer J (2018). Skeletal muscle wasting in chronic heart failure. ESC Heart Fail.

[21] Li W, Yue T, Liu Y (2020). New understanding of the pathogenesis and treatment of stroke-related sarcopenia. Biomed Pharmacother.

[22] Chin SO, Rhee SY, Chon S (2013). Sarcopenia is independently associated with cardiovascular disease in older Korean adults: the Korea National Health and Nutrition Examination Survey (KNHANES) from 2009. PLoS ONE.

[23] Fukuda T, Bouchi R, Takeuchi T (2018). Sarcopenic obesity assessed using dual energy X-ray absorptiometry (DXA) can predict cardiovascular disease in patients with type 2 diabetes: a retrospective observational study. Cardiovasc Diabetol.

[24] Hong SH, Choi KM (2020). Sarcopenic obesity, insulin resistance, and their implications in cardiovascular and metabolic consequences. Int J Mol Sci.

[25] Zhao Y, Hu Y, Smith JP, Strauss J, Yang G (2014). Cohort profile: the China Health and Retirement Longitudinal Study (CHARLS). Int J Epidemiol.

[26] Chen LK, Woo J, Assantachai P (2020). Asian working group for sarcopenia: 2019 consensus update on sarcopenia diagnosis and treatment. J Am Med Dir Assoc.

[27] Delmonico MJ, Harris TB, Lee JS (2007). Alternative definitions of sarcopenia, lower extremity performance, and functional impairment with aging in older men and women. J Am Geriatr Soc.

[28] Alexandre T da S, Duarte Y de O, Santos JLF, Wong R, Lebrão ML (2014). Sarcopenia according to the European working group on sarcopenia in older people (EWGSOP) versus dynapenia as a risk factor for mortality in the elderly. J Nutr Health Aging.

[29] Gao K, Cao LF, Ma WZ (2022). Association between sarcopenia and cardiovascular disease among middle-aged and older adults: findings from the China health and retirement longitudinal study. EClinicalMedicine.

[30] Wu X, Li X, Xu M, Zhang Z, He L, Li Y (2021). Sarcopenia prevalence and associated factors among older Chinese population: findings from the China Health and Retirement Longitudinal Study. PLoS ONE.

[31] Li H, Zheng D, Li Z (2019). Association of depressive symptoms with incident cardiovascular diseases in middle-aged and older Chinese adults. JAMA Netw Open.

[32] Xie W, Zheng F, Yan L, Zhong B (2019). Cognitive decline before and after incident coronary events. J Am Coll Cardiol.

[33] Bello-Chavolla OY, Almeda-Valdes P, Gomez-Velasco D (2018). METS-IR, a novel score to evaluate insulin sensitivity, is predictive of visceral adiposity and incident type 2 diabetes. Eur J Endocrinol.

[34] Er LK, Wu S, Chou HH (2016). Triglyceride glucose-body mass index is a simple and clinically useful surrogate marker for insulin resistance in nondiabetic individuals. PLoS ONE.

[35] Lim J, Kim J, Koo SH, Kwon GC (2019). Comparison of triglyceride glucose index, and related parameters to predict insulin resistance in Korean adults: an analysis of the 2007-2010 Korean National Health and Nutrition Examination Survey. PLoS ONE.

[36] Liu H, Liu J, Liu J, Xin S, Lyu Z, Fu X (2022). Triglyceride to high-density lipoprotein cholesterol (TG/HDL-C) ratio, a simple but effective indicator in predicting type 2 diabetes mellitus in older adults. Front Endocrinol (Lausanne).

[37] Duan M, Zhao X, Li S (2024). Metabolic score for insulin resistance (METS-IR) predicts all-cause and cardiovascular mortality in the general population: evidence from NHANES 2001-2018. Cardiovasc Diabetol.

[38] Salazar MR, Carbajal HA, Espeche WG (2013). Identifying cardiovascular disease risk and outcome: use of the plasma triglyceride/high-density lipoprotein cholesterol concentration ratio versus metabolic syndrome criteria. J Intern Med.

[39] Che B, Zhong C, Zhang R (2023). Triglyceride-glucose index and triglyceride to high-density lipoprotein cholesterol ratio as potential cardiovascular disease risk factors: an analysis of UK biobank data. Cardiovasc Diabetol.

[40] Hong S, Han K, Park CY (2020). The triglyceride glucose index is a simple and low-cost marker associated with atherosclerotic cardiovascular disease: a population-based study. BMC Med.

[41] Li F, Wang Y, Shi B (2024). Association between the cumulative average triglyceride glucose-body mass index and cardiovascular disease incidence among the middle-aged and older population: a prospective nationwide cohort study in China. Cardiovasc Diabetol.

[42] Cui C, Qi Y, Song J (2024). Comparison of triglyceride glucose index and modified triglyceride glucose indices in prediction of cardiovascular diseases in middle aged and older Chinese adults. Cardiovasc Diabetol.

[43] Chai KC, Chen WM, Chen M, Shia BC, Wu SY (2022). Association between preexisting sarcopenia and stroke in patients with type 2 diabetes mellitus. J Nutr Health Aging.

[44] DeFronzo RA, Tripathy D (2009). Skeletal muscle insulin resistance is the primary defect in type 2 diabetes. Diabetes Care.

[45] Kalyani RR, Corriere M, Ferrucci L (2014). Age-related and disease-related muscle loss: the effect of diabetes, obesity, and other diseases. Lancet Diabetes Endocrinol.

[46] Sartori R, Romanello V, Sandri M (2021). Mechanisms of muscle atrophy and hypertrophy: implications in health and disease. Nat Commun.

[47] Li CW, Yu K, Shyh-Chang N (2022). Pathogenesis of sarcopenia and the relationship with fat mass: descriptive review. J Cachexia Sarcopenia Muscle.

[48] Abete I, Konieczna J, Zulet MA (2019). Association of lifestyle factors and inflammation with sarcopenic obesity: data from the PREDIMED-Plus trial. J Cachexia Sarcopenia Muscle.

[49] Bilski J, Pierzchalski P, Szczepanik M, Bonior J, Zoladz JA (2022). Multifactorial mechanism of sarcopenia and sarcopenic obesity. role of physical exercise, microbiota and myokines. Cells.

[50] Kernan WN, Inzucchi SE, Viscoli CM, Brass LM, Bravata DM, Horwitz RI (2002). Insulin resistance and risk for stroke. Neurology (ECronicon).

[51] Kim TN, Choi KM (2015). The implications of sarcopenia and sarcopenic obesity on cardiometabolic disease. J Cell Biochem.

[52] Korytowski W, Wawak K, Pabisz P (2015). Impairment of macrophage cholesterol efflux by cholesterol hydroperoxide trafficking: implications for atherogenesis under oxidative stress. Arterioscler Thromb Vasc Biol.

[53] China Health and Retirement Longitudinal Study [Web page in Chinese]. CHARLS.

