# Supplementary material for: Association of Insulin Resistance, Sarcopenia, and Risk of Cardiovascular Disease: Findings From the China Health and Retirement Longitudinal Study
Source: JMIR Aging. 2025 Dec 31;8:e80115. doi: 10.2196/80115 (PMC12755897; doi:10.2196/80115)
Supplement: Multimedia Appendix 1 [file aging-v8-e80115-s001.docx]

**Table S1.** The independent and adjusted associations of IR surrogate indicators and sarcopenia status with CVD risk.

| Characteristics | Crude model | | Adjusted model | |
| --- | --- | --- | --- | --- |
|  | HR（95%CI） | P | HR（95%CI） | P |
| **IR surrogate indicators^a^** | | | | |
| TyG (per SD) | 1.312(1.186-1.451) | <0.001 | 1.172(1.026-1.339) | 0.020 |
| TyG-BMI (per SD) | 1.006(1.004-1.008) | <0.001 | 1.004(1.001-1.007) | 0.016 |
| TyG-WC (per SD) | 1.002(1.001-1.002) | <0.001 | 1.001(1.000-1.001) | 0.035 |
| TyG-WHtR (per SD) | 1.275(1.174-1.385) | <0.001 | 1.081(0.971-1.204) | 0.156 |
| TG/HDL-C (per SD) | 1.026(0.963-1.092) | 0.428 | 1.015(0.948-1.086) | 0.671 |
| METS-IR (per SD) | 1.024(1.015-1.033) | <0.001 | 1.012(0.998-1.026) | 0.097 |
| **Sarcopenia status** | | | | |
| Non-sarcopenia | 1.00(Reference) |  | 1.00(Reference) |  |
| Possible sarcopenia^b^ | 1.462(1.280-1.670) | <0.001 | 1.313(1.118-1.540) | 0.001 |
| Possible sarcopenia^c^ | 1.462(1.280-1.670) | <0.001 | 1.303(1.110-1.530) | 0.001 |
| Possible sarcopenia^d^ | 1.462(1.280-1.670) | <0.001 | 1.296(1.104-1.523) | 0.002 |
| Possible sarcopenia^e^ | 1.462(1.280-1.670) | <0.001 | 1.303(1.109-1.531) | 0.001 |
| Possible sarcopenia^f^ | 1.462(1.280-1.670) | <0.001 | 1.321(1.126-1.551) | 0.001 |
| Possible sarcopenia^g^ | 1.462(1.280-1.670) | <0.001 | 1.312(1.117-1.540) | 0.001 |
| Sarcopenia^b^ | 1.171(0.853-1.608) | 0.330 | 1.197(0.801-1.787) | 0.380 |
| Sarcopenia^c^ | 1.171(0.853-1.608) | 0.330 | 1.297(0.862-1.951) | 0.212 |
| Sarcopenia^d^ | 1.171(0.853-1.608) | 0.330 | 1.233(0.824-1.846) | 0.308 |
| Sarcopenia^e^ | 1.171(0.853-1.608) | 0.330 | 1.205(0.806-1.801) | 0.364 |
| Sarcopenia^f^ | 1.171(0.853-1.608) | 0.330 | 1.182(0.792-1.765) | 0.413 |
| Sarcopenia^g^ | 1.171(0.853-1.608) | 0.330 | 1.243(0.829-1.866) | 0.293 |
| Severe sarcopenia^b^ | 1.311(0.878-1.957) | 0.185 | 1.713(1.078-2.721) | 0.023 |
| Severe sarcopenia^c^ | 1.311(0.878-1.957) | 0.185 | 1.879(1.174-3.005) | 0.009 |
| Severe sarcopenia^d^ | 1.311(0.878-1.957) | 0.185 | 1.751(1.101-2.783) | 0.018 |
| Severe sarcopenia^e^ | 1.311(0.878-1.957) | 0.185 | 1.725(1.086-2.742) | 0.021 |
| Severe sarcopenia^f^ | 1.311(0.878-1.957) | 0.185 | 1.711(1.077-2.719) | 0.023 |
| Severe sarcopenia^g^ | 1.311(0.878-1.957) | 0.185 | 1.795(1.125-2.863) | 0.014 |

Adjusted model: adjusted for Age, Sex, Smoker, Ex-smoker, Drinking, Hypertension, Diabetes, Dyslipidemia, and obesity.

Sarcopenia status is classified into four categories (Non-sarcopenia; Possible sarcopenia; Sarcopenia; Severe sarcopenia).

^a^Further adjusted for sarcopenia status.

^b-g^Further adjusted for TyG (per SD), TyG-BMI (per SD), TyG-WC (per SD), TyG-WHtR (per SD), TG/HDL-C (per SD), METS-IR (per SD), respectively.

**Table S2.** Additive interaction analysis of sarcopenia and IR surrogate indicators with CVD risk (median cutoffs).

| **Sarcopenia status** | **IR-Indicator** | **RERI_95%CI** | **AP** | **S** | **N-both** |
| --- | --- | --- | --- | --- | --- |
| Possible sarcopenia | TyG | 0.045 (-0.476-0.567) | 0.029 | 1.089 | 685 |
|  | TyG-BMI | -0.049 (-0.657-0.558) | -0.032 | 0.914 | 714 |
|  | TyG-WC | -0.395 (-0.954-0.163) | -0.295 | 0.463 | 753 |
|  | TyG-WHtR | -0.433 (-0.978-0.113) | -0.346 | 0.368 | 761 |
|  | TG/HDL-C | 0.139 (-0.396-0.674) | 0.083 | 1.263 | 690 |
|  | METS-IR | 0.039 (-0.546-0.624) | 0.025 | 1.075 | 718 |
| Sarcopenia | TyG | 0.169 (-0.827-1.165) | 0.122 | 1.795 | 59 |
|  | TyG-BMI | -1.317 (-1.826--0.809) | -309320.123^+^ | -3.152 | 1 |
|  | TyG-WC | -0.578 (-1.879-0.722) | -0.943 | -2.010 | 13 |
|  | TyG-WHtR | -0.643 (-1.521-0.235) | -1.244 | -3.026 | 24 |
|  | TG/HDL-C | 0.315 (-0.842-1.471) | 0.195 | 2.053 | 48 |
|  | METS-IR | -1.356 (-1.863--0.849) | -243761.961^+^ | -2.810 | 2 |
| Severe sarcopenia | TyG | -0.328 (-1.823-1.166) | -0.207 | 0.642 | 44 |
|  | TyG-BMI | -1.863 (-2.645--1.081) | -419217.933^+^ | -1.159 | 2 |
|  | TyG-WC | -0.154 (-2.054-1.745) | -0.102 | 0.767 | 14 |
|  | TyG-WHtR | -0.072 (-1.543-1.399) | -0.049 | 0.869 | 33 |
|  | TG/HDL-C | -0.457 (-2.017-1.102) | -0.284 | 0.571 | 36 |
|  | METS-IR | -1.891 (-2.673--1.11) | -425758.337^+^ | -1.122 | 1 |

CVD, cardiovascular disease; IR, Insulin resistance; RERI, relative excess risk due to interaction; AP, attributable proportion due to interaction; S, synergy index; N_both, both conditions present (possible sarcopenia/sarcopenia/severe sarcopenia & high IR).

^+^Extreme values of AP and S indices may result from small sample sizes or instability in hazard ratio and estimates should be interpreted with caution. Interpretation of additive interaction measures: RERI > 0 indicates synergistic interaction (combined effect greater than the sum of individual effects);

*RERI = 0 indicates no additive interaction*; RERI < 0 indicates antagonistic interaction (combined effect less than the sum of individual effects); AP > 0 indicates the proportion of disease risk attributable to interaction; S > 1 indicates synergistic interaction.

**Table S3.** Additive interaction analysis of sarcopenia and IR surrogate indicators with CVD risk (ROC cutoffs).

| **Sarcopenia status** | **IR surrogate indicators** | **RERI_95%CI (ROC)** | **AP (ROC)** | **S (ROC)** | **N_both (ROC)** |
| --- | --- | --- | --- | --- | --- |
| Possible sarcopenia | TyG | -0.033 (-0.572-0.507) | -0.021 | 0.946 | 715 |
|  | TyG-BMI | -0.097 (-0.744-0.551) | -0.059 | 0.868 | 781 |
|  | TyG-WC | 0.016 (-0.595-0.627) | 0.009 | 1.021 | 454 |
|  | TyG-WHtR | -0.328 (-0.878-0.222) | -0.233 | 0.555 | 611 |
|  | TG/HDL-C | 0.143 (-0.408-0.695) | 0.082 | 1.240 | 671 |
|  | METS-IR | 0.074 (-0.538-0.685) | 0.044 | 1.123 | 700 |
| Sarcopenia | TyG | 0.295 (-0.725-1.315) | 0.198 | 2.538 | 63 |
|  | TyG-BMI | -1.461 (-1.994--0.928) | -325475.206^+^ | -2.168 | 1 |
|  | TyG-WC | NA (NA-NA)* | NA* | NA* | 0 |
|  | TyG-WHtR | -1.352 (-1.859--0.845) | -1611706.477^+^ | -2.841 | 12 |
|  | TG/HDL-C | 0.394 (-0.837-1.624) | 0.226 | 2.130 | 45 |
|  | METS-IR | -1.471 (-1.994--0.949) | -259684.893^+^ | -2.122 | 2 |
| Severe sarcopenia | TyG | -0.391 (-1.902-1.12) | -0.246 | 0.601 | 45 |
|  | TyG-BMI | -2.077 (-2.915--1.239) | -408977.666^+^ | -0.929 | 3 |
|  | TyG-WC | -2.106 (-2.888--1.323) | -417883.710^+^ | -0.904 | 2 |
|  | TyG-WHtR | 0.057 (-1.863-1.977) | 0.032 | 1.081 | 20 |
|  | TG/HDL-C | -0.506 (-2.096-1.085) | -0.308 | 0.560 | 36 |
|  | METS-IR | -2.011 (-2.808--1.215) | -444265.675 | -0.989 | 1 |

CVD, cardiovascular disease; IR, Insulin resistance; RERI, relative excess risk due to interaction; AP, attributable proportion due to interaction; S, synergy index; N_both, both conditions present (possible sarcopenia/sarcopenia/severe sarcopenia & high IR).

*NA values indicate that the model failed to converge due to insufficient sample size in the "both conditions" group (N_both = 0)*.

^+^Extreme values of AP and S indices may result from small sample sizes or instability in hazard ratio and estimates should be interpreted with caution. Interpretation of additive interaction measures:

RERI > 0 indicates synergistic interaction (combined effect greater than the sum of individual effects); *RERI = 0 indicates no additive interaction*; RERI < 0 indicates antagonistic interaction (combined effect less than the sum of individual effects); AP > 0 indicates the proportion of disease risk attributable to interaction; S > 1 indicates synergistic interaction.

**Table S4.** Multiplicative interaction analysis of sarcopenia and IR surrogate indicators with CVD risk (median cutoffs).

| **Sarcopenia status** | **IR surrogate indicators** | **Interaction_HR** | **P_Value** | **N-both** |
| --- | --- | --- | --- | --- |
| Possible sarcopenia | TyG | 0.988 (0.724-1.347) | 0.938 | 685 |
|  | TyG-BMI | 0.921 (0.672-1.262) | 0.609 | 714 |
|  | TyG-WC | 0.728 (0.533-0.995) | 0.046 | 753 |
|  | TyG-WHtR | 0.715 (0.523-0.979) | 0.036 | 761 |
|  | TG/HDL-C | 1.044 (0.764-1.428) | 0.786 | 690 |
|  | METS-IR | 0.983 (0.717-1.346) | 0.913 | 718 |
| Sarcopenia | TyG | 1.145 (0.527-2.486) | 0.733 | 59 |
|  | TyG-BMI | 0 (0-Inf)* | 0.989 | 1 |
|  | TyG-WC | 0.511 (0.069-3.798) | 0.512 | 13 |
|  | TyG-WHtR | 0.443 (0.104-1.892) | 0.272 | 24 |
|  | TG/HDL-C | 1.252 (0.568-2.76) | 0.578 | 48 |
|  | METS-IR | 0 (0-Inf)* | 0.984 | 2 |
| Severe sarcopenia | TyG | 0.762 (0.311-1.865) | 0.552 | 44 |
|  | TyG-BMI | 0 (0-Inf)* | 0.988 | 2 |
|  | TyG-WC | 0.872 (0.253-3.008) | 0.828 | 14 |
|  | TyG-WHtR | 0.941 (0.358-2.475) | 0.902 | 33 |
|  | TG/HDL-C | 0.694 (0.276-1.744) | 0.437 | 36 |
|  | METS-IR | 0 (0-Inf)* | 0.988 | 1 |

CVD, cardiovascular disease; IR, Insulin resistance; N_both, both conditions present (possible sarcopenia/sarcopenia/severe sarcopenia & high IR); HR estimates of 0.000 indicate that the multiplicative interaction term could not be reliably estimated due to extremely small sample sizes in the "both conditions" group (n_both ≤ 3).

A multiplicative interaction HR significantly different from 1 indicates that the combined effect of sarcopenia and high IR on CVD risk differs from the product of their individual effects. HR < 1 suggests the combined effect is less than multiplicative; HR > 1 suggests the combined effect is greater than multiplicative.

**Table S5.** Multiplicative interaction analysis of sarcopenia and IR surrogate indicators with CVD risk (ROC cutoffs).

| **Sarcopenia status** | **IR surrogate indicators** | **Interaction_HR** | **P_Value** | **N-both** |
| --- | --- | --- | --- | --- |
| Possible sarcopenia | TyG | 0.928 (0.678-1.269) | 0.638 | 715 |
|  | TyG-BMI | 0.877 (0.631-1.217) | 0.431 | 781 |
|  | TyG-WC | 0.939 (0.689-1.28) | 0.689 | 454 |
|  | TyG-WHtR | 0.756 (0.557-1.025) | 0.072 | 611 |
|  | TG/HDL-C | 1.035 (0.758-1.414) | 0.829 | 671 |
|  | METS-IR | 0.991 (0.724-1.356) | 0.955 | 700 |
| Sarcopenia | TyG | 1.264 (0.587-2.718) | 0.549 | 63 |
|  | TyG-BMI | 0 (0-Inf)* | 0.989 | 1 |
|  | TyG-WC | NA (NA-NA)* | NA* | 0 |
|  | TyG-WHtR | 0 (0-Inf)* | 0.985 | 12 |
|  | TG/HDL-C | 1.304 (0.592-2.875) | 0.510 | 45 |
|  | METS-IR | 0 (0-Inf)* | 0.984 | 2 |
| Severe sarcopenia | TyG | 0.73 (0.298-1.788) | 0.492 | 45 |
|  | TyG-BMI | 0 (0-Inf)* | 0.983 | 3 |
|  | TyG-WC | 0 (0-Inf)* | 0.983 | 2 |
|  | TyG-WHtR | 0.977 (0.323-2.949) | 0.966 | 20 |
|  | TG/HDL-C | 0.67 (0.267-1.683) | 0.394 | 36 |
|  | METS-IR | 0 (0-Inf)* | 0.988 | 1 |

IR, Insulin resistance; N_both, both conditions present (possible sarcopenia/sarcopenia/severe sarcopenia & high IR); NA values and HR estimates of 0.000 indicate that the multiplicative interaction term could not be reliably estimated due to extremely small sample sizes in the "both conditions" group (n_both ≤ 3).

Interpretation: A multiplicative interaction HR significantly different from 1 indicates that the combined effect of sarcopenia and high IR on CVD risk differs from the product of their individual effects. HR < 1 suggests the combined effect is less than multiplicative; HR > 1 suggests the combined effect is greater than multiplicative.

**Table S6.** ROC analysis of IR surrogate indicators sarcopenia-related traits and for CVD risk stratification.

| **Indicator** | **Optimal Cutoff** | **Sensitivity** | **Specificity** | **AUC** |
| --- | --- | --- | --- | --- |
| TyG | 8.564 | 0.602 | 0.501 | 0.558 |
| TyG-BMI | 192.470 | 0.659 | 0.464 | 0.576 |
| TyG-WC | 805.334 | 0.382 | 0.748 | 0.572 |
| TyG-WHtR | 2.090 | 0.588 | 0.534 | 0.557 |
| TG/HDL-C | 4.914 | 0.478 | 0.642 | 0.573 |
| METS-IR | 34.260 | 0.582 | 0.540 | 0.570 |
| Handgrip Strength (kg) | 26.850 | 0.487 | 0.587 | 0.541 |
| Chair Stand Test Time (s) | 9.405 | 0.589 | 0.509 | 0.556 |
| 5-meter Gait Speed Time (s) | 3.535 | 0.420 | 0.612 | 0.514 |
| ASM Index (kg/m²) | 6.642 | 0.493 | 0.547 | 0.503 |

CVD, cardiovascular disease; IR, Insulin resistance; AUC, area under the receiver operating characteristic curve; TyG, triglyceride-glucose index; BMI, body mass index; WC, waist circumference; WHtR, waist-to-height ratio; TG/HDL-C, triglyceride to high-density lipoprotein cholesterol ratio; METS-IR, metabolic score for insulin resistance; ASM, appendicular skeletal muscle.

Cutoff values were determined by maximizing Youden's index (sensitivity + specificity - 1). AUC interpretation: 0.5 = no discrimination, 0.5-0.7 = poor to fair, 0.7-0.8 = acceptable, 0.8-0.9 = excellent, >0.9 = outstanding.
